# Supplementary material for: TDP2 suppresses chromosomal translocations induced by DNA topoisomerase II during gene transcription
Source: Nat Commun. 2017 Aug 10;8:233. doi: 10.1038/s41467-017-00307-y (PMC5550487; doi:10.1038/s41467-017-00307-y)
Supplement: Supplementary file 1 — Supplementary Information [file 41467_2017_307_MOESM1_ESM.pdf]

File Name: Supplementary Information
Description: Supplementary Figures and Supplementary Tables

File Name: Peer Review File
Description:

Cell Type	treatment	No. Cells	Chromos-type (freq. per cell)			Chromatid-type (freq. per cell)				
			Exchange	Breaks	**Total**	Exchange	Breaks	Gaps	**Total**	**Total**
**normal**	DMSO	49	0.08	0	**0.08**	0.02	0.06	0.02	**0.10**	**0.18**
**normal**	Etop.	50	0.04	0.08	**0.12** ns	0.08	0.16	0.04	**0.28** ns	**0.40** ns
**patient**	DMSO	50	0.06	0.04	**0.10** ***	0	0.02	0.02	**0.04** ***	**0.14** ***
**patient**	Etop.	53	0.13	0.30	**0.43** ***	0.36	2.83	0.34	**3.53** ***	**3.96** ***

Supplementary Figure 1. Chromosome and chromatid type aberrations in normal and TDP2 patient lymphoblastoid cells. Chromosome aberrations were scored by M-FISH karyotyping of control and TDP2 patient cells following continuous treatment with 50nM etoposide for 20 hr. Frequencies are the total number of the indicated event per metaphase. Statistical significance by ANOVA (Tukey simultaneous test). *ns*, not significant; *** P<0.005.

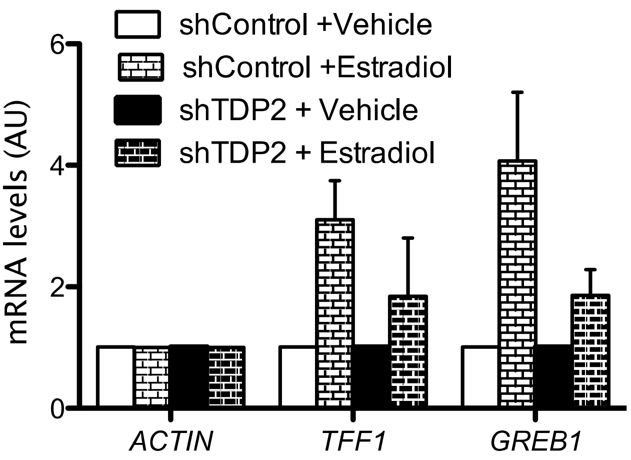

Supplementary Figure 2. ER-dependent gene induction by 17ß-estradiol. mRNA levels for two ER-responsive genes in mock-depleted (Mock KD) or TDP2-depleted (TDP2 KD) MCF7 cells before and 12 hours after stimulation with 100nM 17ß-estradiol. mRNA levels were quantified by qRT-PCR and are expressed as arbitrary units (AU) normalized first to *ACTB* levels under the same experimental condition and then to the levels of the relevant gene of interest under control conditions without hormone. Data are the mean (± s.e.m.) of four independent experiments.

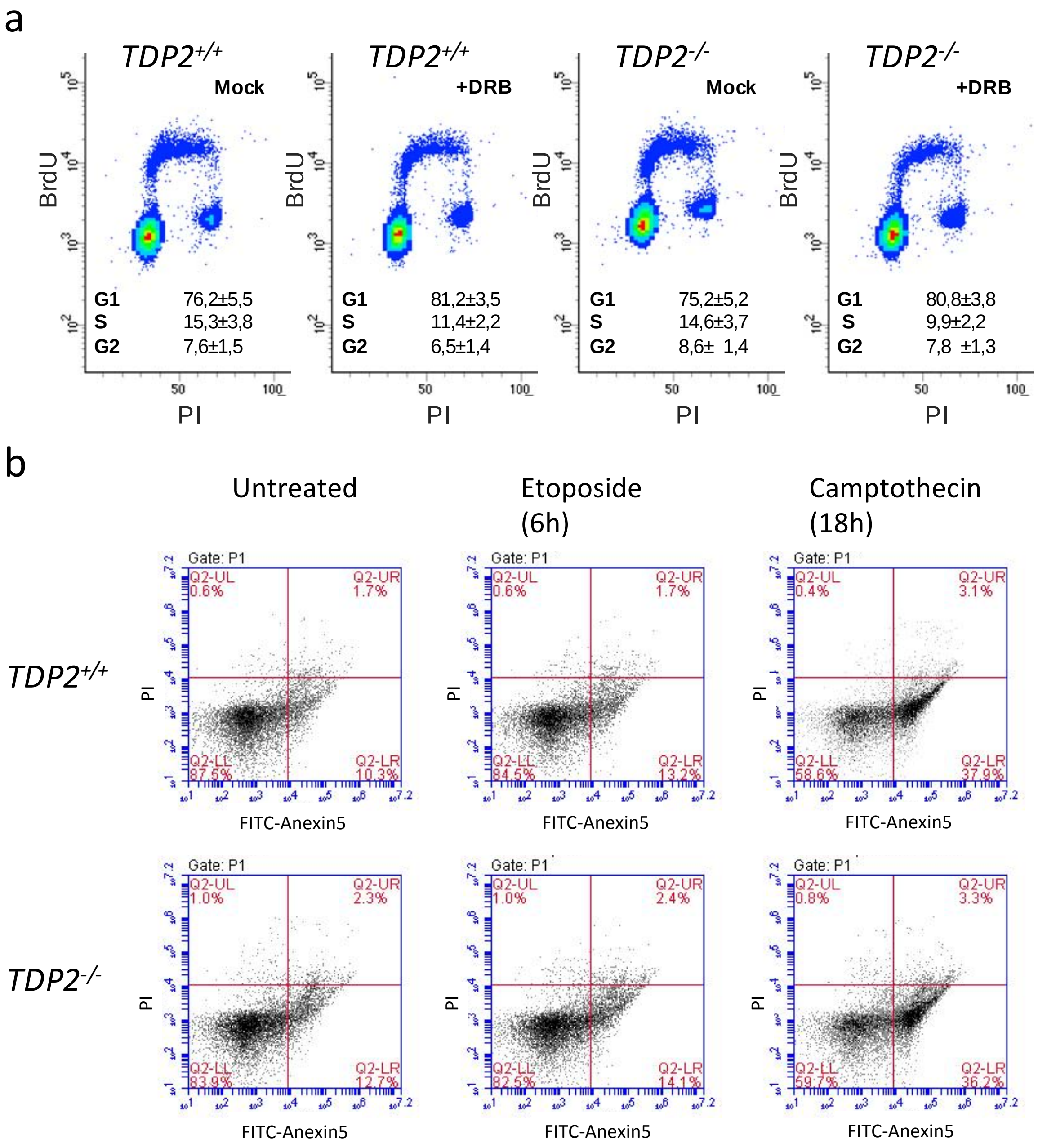

Supplementary Figure 3. FACS analysis of exponentially growing wild type and *TDP2*$^{-/-}$ RPE-1 cells. **a.** Cells were mock-treated (DMSO) or treated with DRB (100µM) for 3 hr. Cells were pulse labelled for 15 min with BrdU for subsequent cell cycle analysis. Data are the mean (± s.e.m.) of three independent experiments. **b.** Cells were mock-treated (DMSO) or treated with etoposide (100µM) for 6 hr. Cells were trypsinized and phosphatidylserine exclusion (as a marker of early apoptosis) measured by Anexin5 (FITC) staining. Cells treated with camptothecin (10µM for 18 hr) were employed as a positive control.

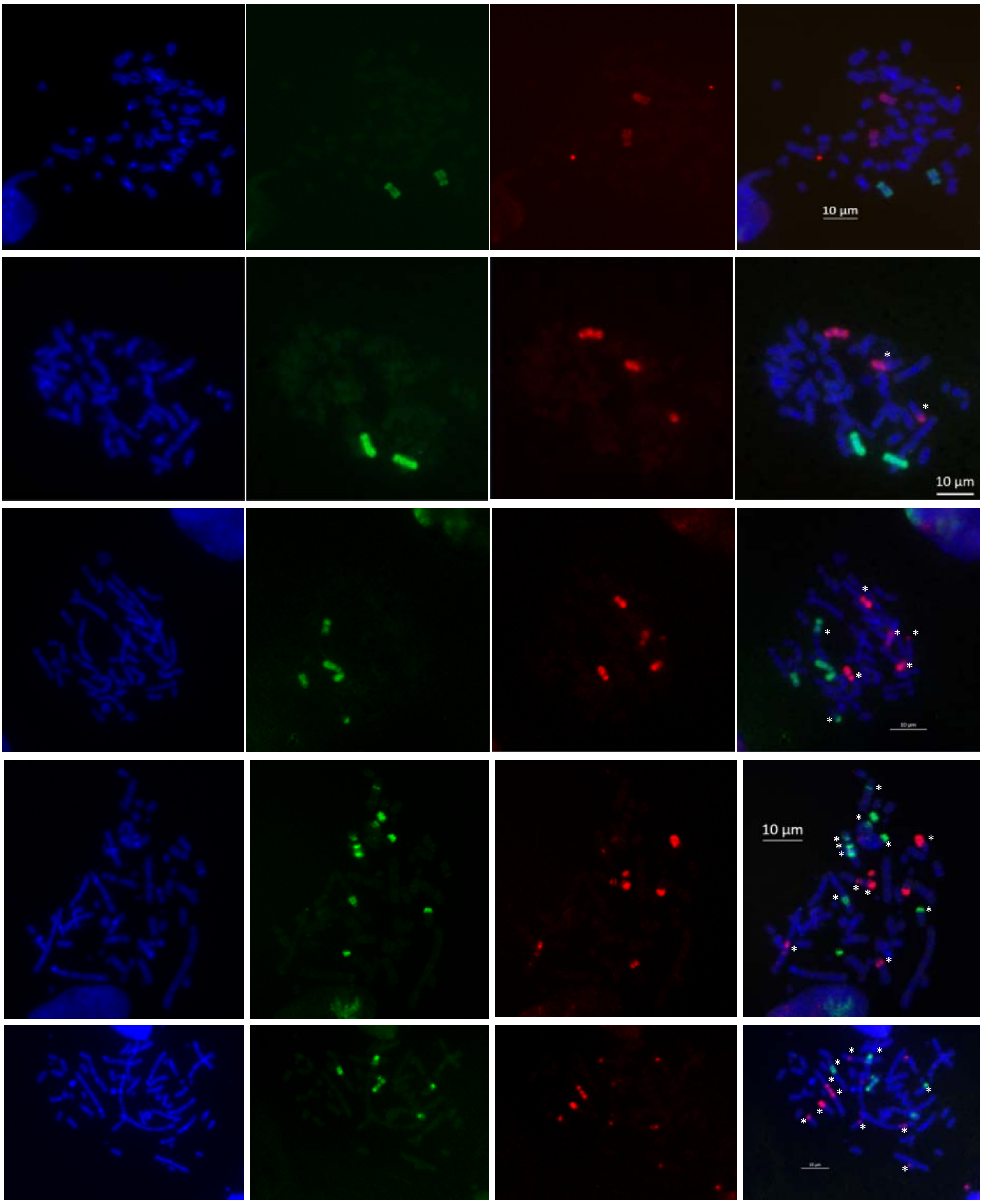

Supplementary Figure 4. Representative chromosome spreads scored in *Fig.4a*. Translocations in Chromosomes 8 (green probe) or 11 (red probe) are indicated with white asterisks. Scale bars 10µM. A metaphase spread without Chr8 or Chr 11 translocations is shown in the top panel.

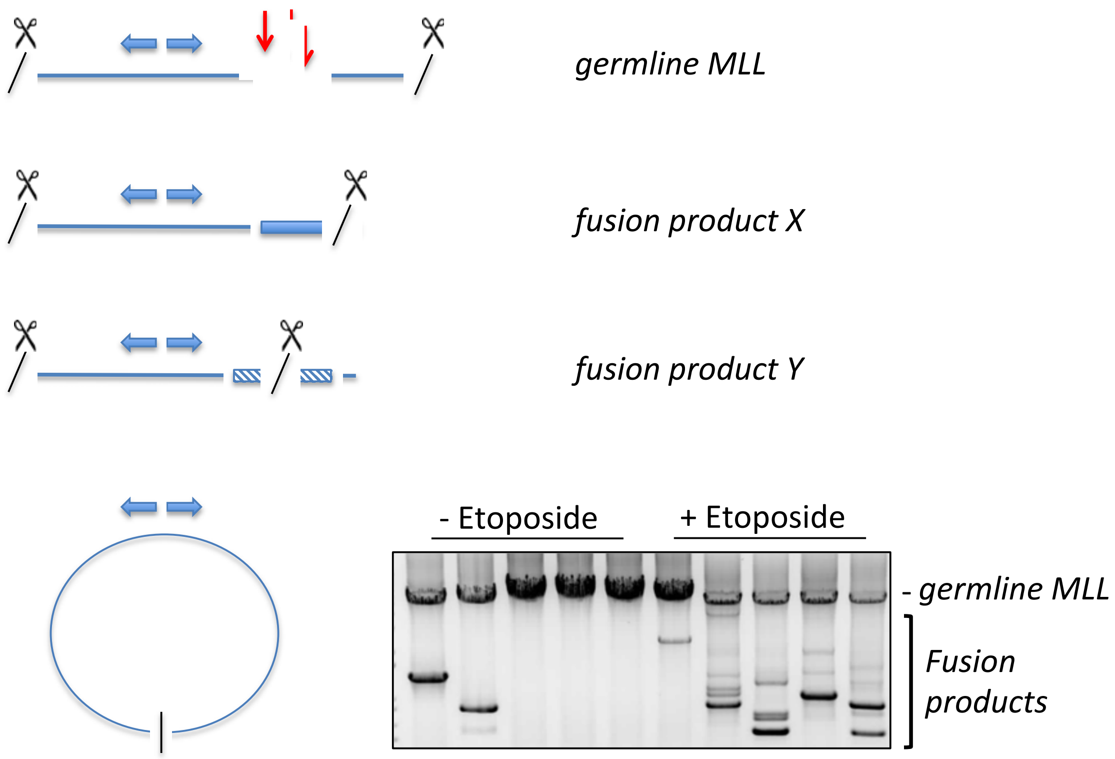

Supplementary Figure 5. Inverse PCR strategy for the isolation of *MLL* translocation partners. Red arrows indicate the *MLL* BCR hotspot for translocatons. Each lane is a single PCR from 40ng of genomic DNA processed as indicated (see experimental procedures for details).

Fig.2A

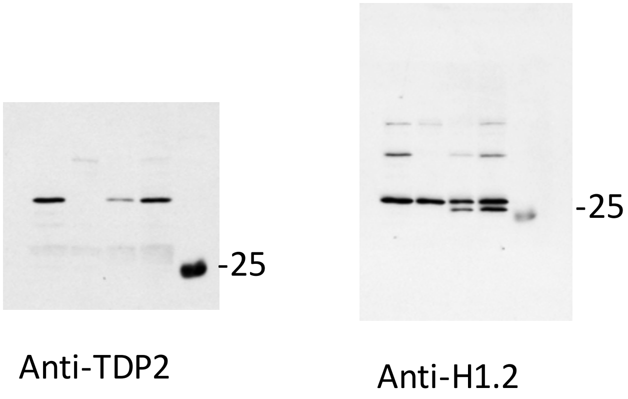

Fig.2D

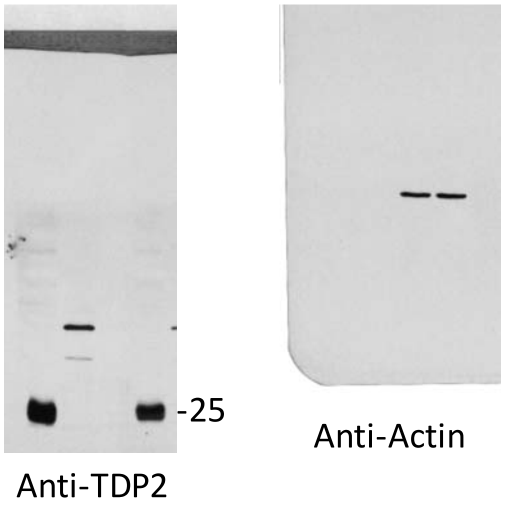

Fig.3D

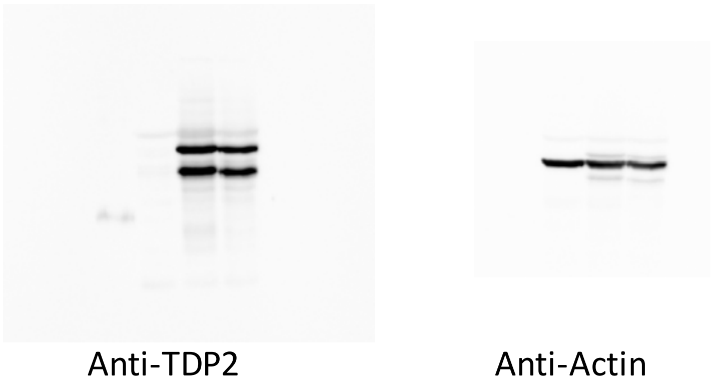

Supplementary Figure 6. Full blots from which the indicated figure panels were generated

2.1. Total aberrations

	TDP2	-/-	-/-	+/+	+/+
TDP2	**Etop.**	**0**	**50**	**0**	**50**
-/-	-	-	< 0.001	0.994	0.421
-/-	+	< 0.001	-	< 0.001	< 0.001
+/+	-	0.994	< 0.001	-	0.585
+/+	+	0.421	< 0.001	0.585	-

2.2. Total chromosome aberrations

	TDP2	-/-	-/-	+/+	+/+
TDP2	**Etop.**	**0**	**50**	**0**	**50**
-/-	-	-	0.002	0.995	0.993
-/-	+	0.002	-	0.001	0.005
+/+	-	0.995	0.001	-	0.956
+/+	+	0.993	0.005	0.956	-

2.3. Total chromatid aberrations

	TDP2	-/-	-/-	+/+	+/+
TDP2	**Etop.**	**0**	**50**	**0**	**50**
-/-	-	-	< 0.001	0.984	0.496
-/-	+	< 0.001	-	< 0.001	< 0.001
+/+	-	0.984	< 0.001	-	0.727
+/+	+	0.496	< 0.001	0.727	-

2.4. Total abnormal cells

	TDP2	-/-	-/-	+/+	+/+
TDP2	**Etop.**	**0**	**50**	**0**	**50**
-/-	-	-	< 0.001	0.993	0.356
-/-	+	< 0.001	-	< 0.001	< 0.001
+/+	-	0.993	< 0.001	-	0.523
+/+	+	0.356	< 0.001	0.523	-

Supplementary Table 1. Pairwise p values for comparison of ANOVA levels (Tukey simultaneous test)

Band	Enzyme	*Sample*	*Location*	Cytogenetic Band	bp in junction	Junction
1	Bgl2	*TDP2 +/+*	*TTC7A*	2p21	**4**	ACTCAGGAGAGGGCAC*ACAG*ACTATAGTAATTTCAT
2	Bgl2	*TDP2 +/+*	*GRAMD1B*	11q24.1	**2**	ACTCAGGAACCTACTCAG*TG*ACCAAAGCTCTCCCAGCG
3	Bgl2	*TDP2 -/-*	*ST6GALNAC5*	1p31.1	**0**	GTTTCACCCTCCTTAAAAGT*TAGTTGTTCAATTCATATTG
4	Bgl2	*TDP2 +/+*	*SNX32*	11q13.1	**1**	AGGCAGGCCAGCATAGGCT*G*TATCACCCAAACAAAATGG
5	Bgl2	*TDP2 +/+*	*NPTN*	15q24.1	**0**	TCATTAGATTCCTTATTTTC*TTGTTCAATTCATATTGAAT
6	Bgl2	*TDP2 +/+*	*PVRL1*	11q23.3	**4**	AGTTGGGGGTCTTGCC*TAGG*AATGTTTCATTATGTA
7	Bgl2	*TDP2 +/+*	*RAB11FIP3*	16p13.3	?	
8	Bgl2	*TDP2 -/-*	*FAT4*	4q28.1	?	
9	Bgl2	*TDP2 -/-*	*PMFBP1*	16q22.2	?	
10	Bgl2	*TDP2 -/-*	*CNTN5*	11q22.1	**2**	GTAGTGCCTGGCATCAGG*AA*TAGCATCCCTTTTAATAG
11	Bgl2	*TDP2 -/-*	*ARHGAP32*	11q24.3	**1**	GAAGCAGCATGCCACTGCA*A*TTTCTTTCCACCAACAGAA
12	Bgl2	*TDP2 -/-*	*SLC35F3*	1q42.2	?	
13	Bgl2	*TDP2 -/-*	*Intergenic (CUL5/ACAT1)*	11q22.3	?	
14	SacI	*TDP2 +/+*	*C1orf127*	1p36.22	?	
15	SacI	*TDP2 +/+*	*LOC105369428*	11q14.3	?	
16	SacI	*TDP2 +/+*	*KMT2A*	11q23.3	?	
17	SacI	*TDP2 +/+*	*Intergenic (GRIP1 promoter)*	12q14.3	?	
18	SacI	*TDP2 +/+*	*CNTN6*	3p26.3	**3**	CTCTCACTAGGTGAAGG*ATT*CTCTGTACAACTCATAG
19	SacI	*TDP2 +/+*	*ATP8B4*	15q21.2	?	
20	SacI	*TDP2 -/-*	*CDKL44*	2p22.1	?	
21	SacI	*TDP2 -/-*	*Intergenic (DPY19L2/LOC100418730)*	12q14.2	?	
22	SacI	*TDP2 +/+*	*C11orf57*	11q23.1	**2**	CGGCAGTGGCCTGGGCCA*CA*CTGGTCATGCTTTAGGAG
23	SacI	*TDP2 -/-*	*Intergenic*	16q21	**3**	ATTTTCAGGAATTTGGG*GAG*GGGTACTTAGAATTCTC
24	Bgl2	*TDP2 +/+*	*Intergenic*	17p13.1	?	
25	Bgl2	*TDP2 -/-*	*ZNF609*	15q22.31	**0**	TTCTCCAGAGCCCTTCTCAC*GTAATTTCATCCACAGAAAA
26	Bgl2	*TDP2 +/+*	*ncRNA XR_251278.2*	2q35	?	
27	Bgl2	*TDP2 +/+*	*ncRNA XR_918087.1*	1q25.1	**5**	ATGTGGTAGTGGCAAA*GAATC*ATCATGGTTTGATAC
28	*Bgl2*	*TDP2 -/-*	*Non-confirmed gene LOC105369266*	16p11.2	?	
29	Bgl2	*TDP2 +/+*	*Intergenic*	4p15.1	?	
30	Bgl2	*TDP2 +/+*	*SVPE1*	9q31.3	**0**	AGGGGTTTGGAGCTAATGAA*ACCCTGGGTGTTATAGGAAC
31	Bgl2	*TDP2 -/-*	*NRXN1*	2p16.3	?	
32	Bgl2	*TDP2 -/-*	*Intergenic*	2p11.1	?	
33	Bgl2	*TDP2 -/-*	*Intergenic*	3p24.1	?	
34	Bgl2	*TDP2 -/-*	*KMT2A*	11q23.3	**2**	CCTCAGCTTCTCAAGAGT*AG*TCAGTTCACAAACAGTGA
35	Bgl2	*TDP2 +/+*	*Intergenic*	5q11.2	**0**	GATTCCACATGAAGTTAAGG*GAAGTCAAGATTCCTAAGCC
36	Bgl2	*TDP2 +/+*	*COL19A1*	6q13	?	
37	Bgl2	*TDP2 +/+*	*HNRNPUL2*	11q12.3	?	
38	Bgl2	*TDP2 +/+*	*Intergenic*	5p14.1	?	
39	Bgl2	*TDP2 +/+*	*LOC101927902*	7q22.3	?	
40	Bgl2	*TDP2 -/-*	*PDGFC*	4q32.1	?	
41	Bgl2	*TDP2 -/-*	*Intergenic FOSB/RTN2*	19q13.32	?	
42	Bgl2	*TDP2 -/-*	*Intergenic*	14q32.12	?	
43	Bgl2	*TDP2 -/-*	*FGD4*	12p11.21	?	

Supplementary Table 2. **Distribution of genomic translocations detected by iPCR**. Cytogenetic bands were assigned by *GeneCards Human Gene Database. SNX32* and *NPTN*, together with the *MLL* partner, were isolated from a single PCR band in which two translocation events had happened.
